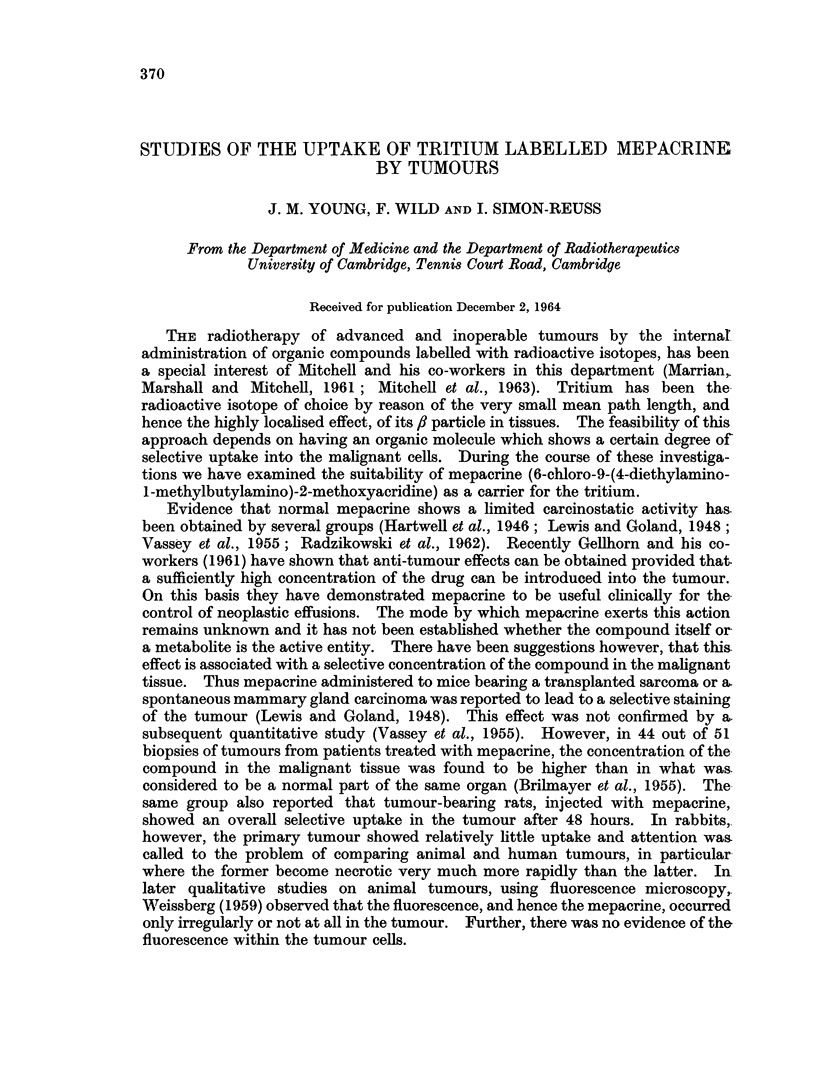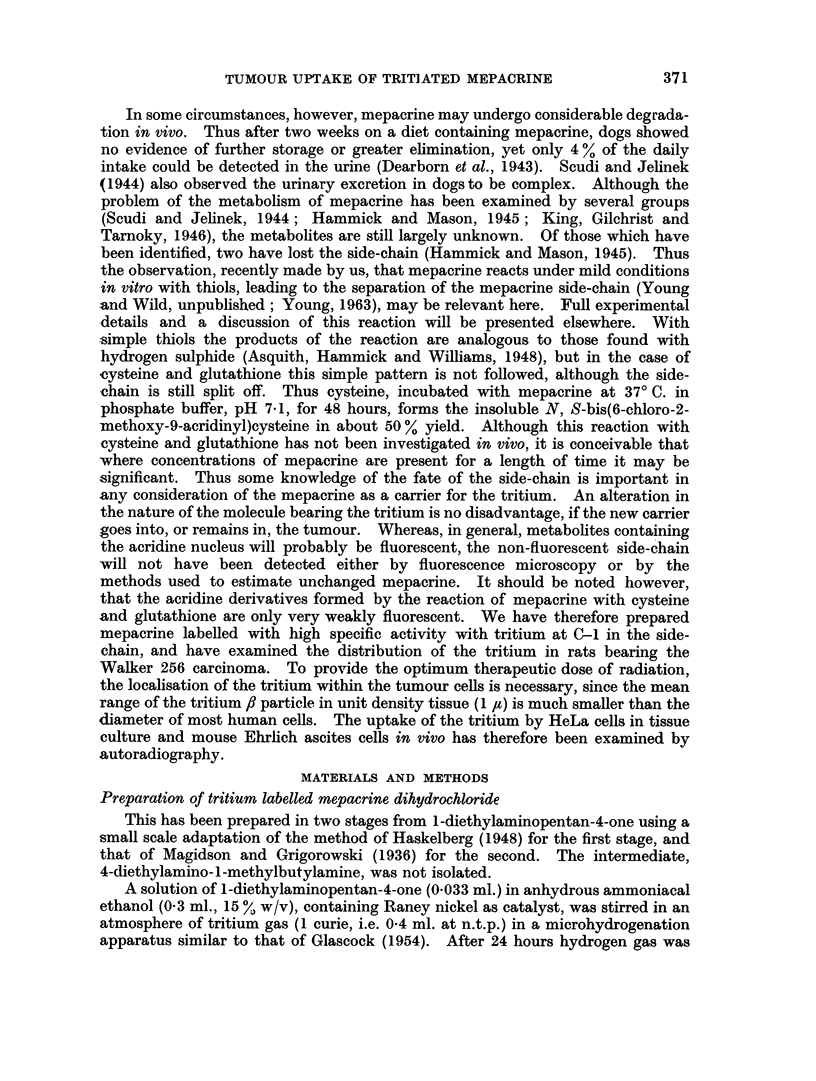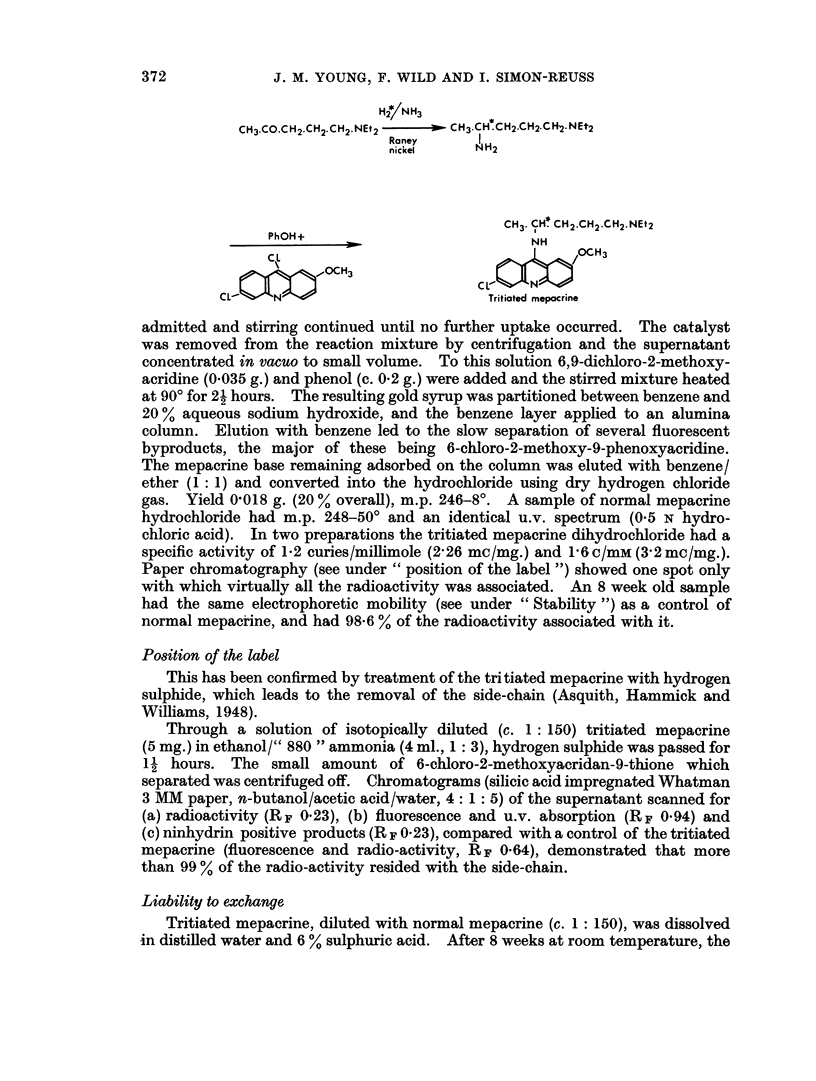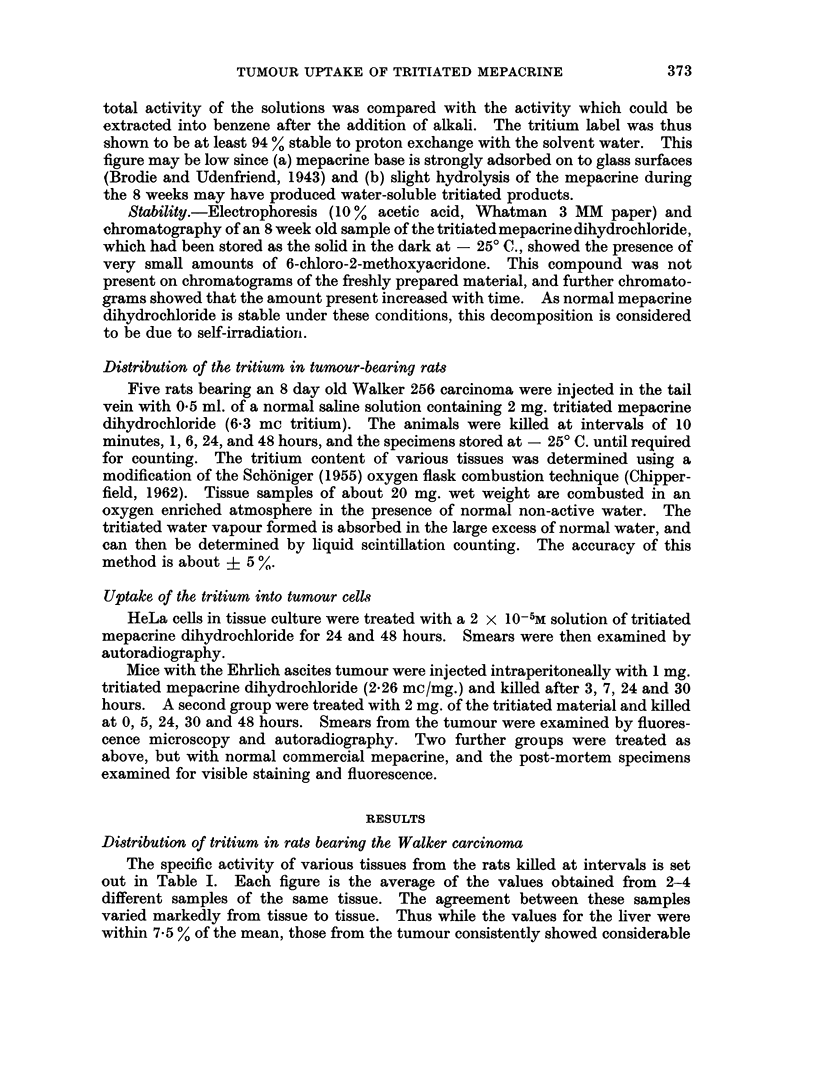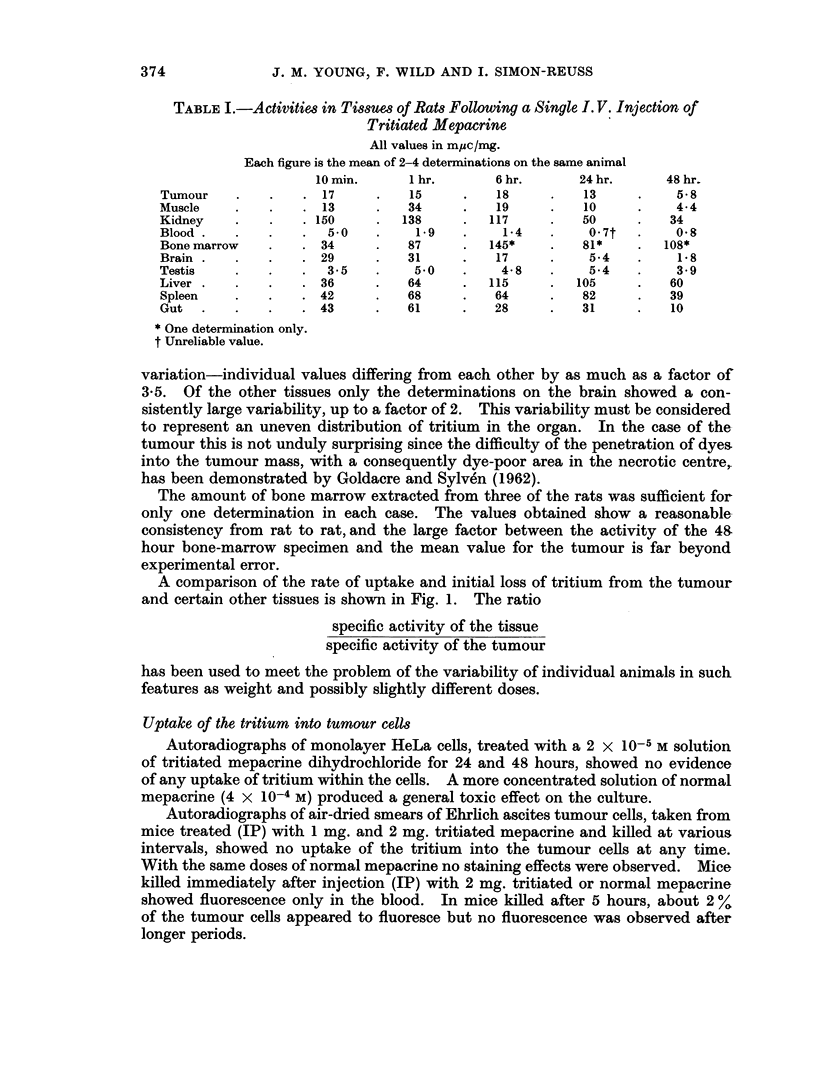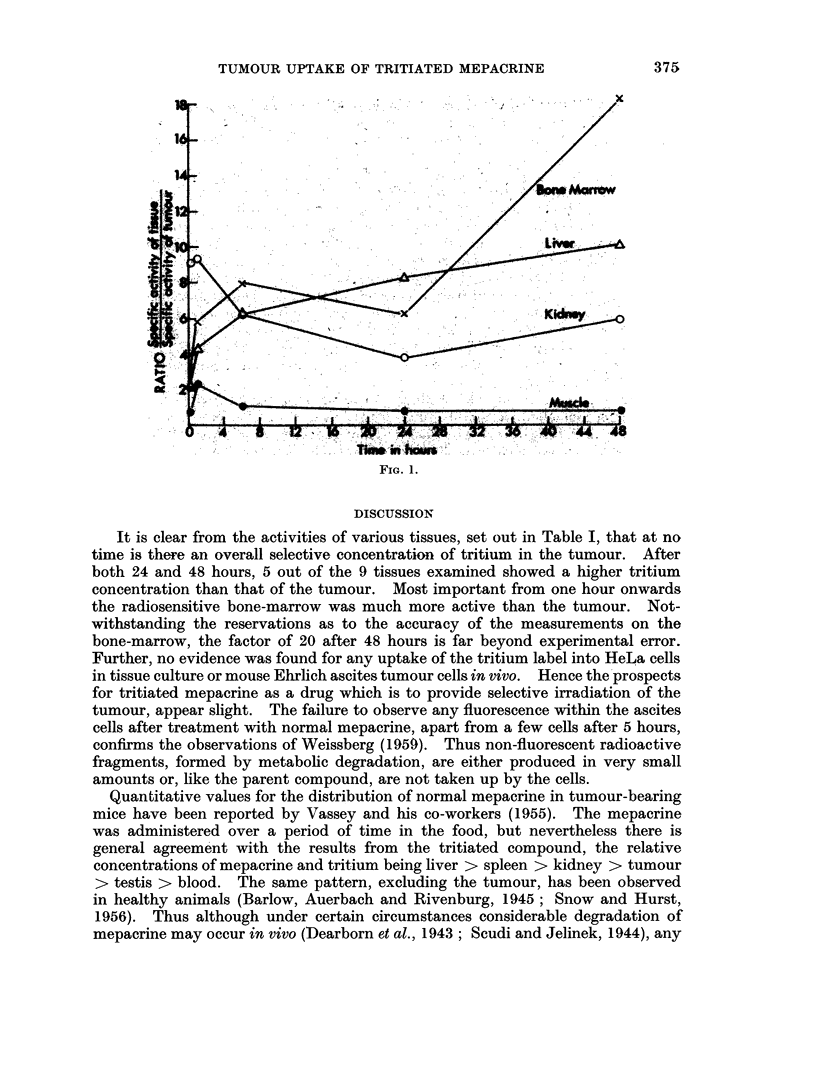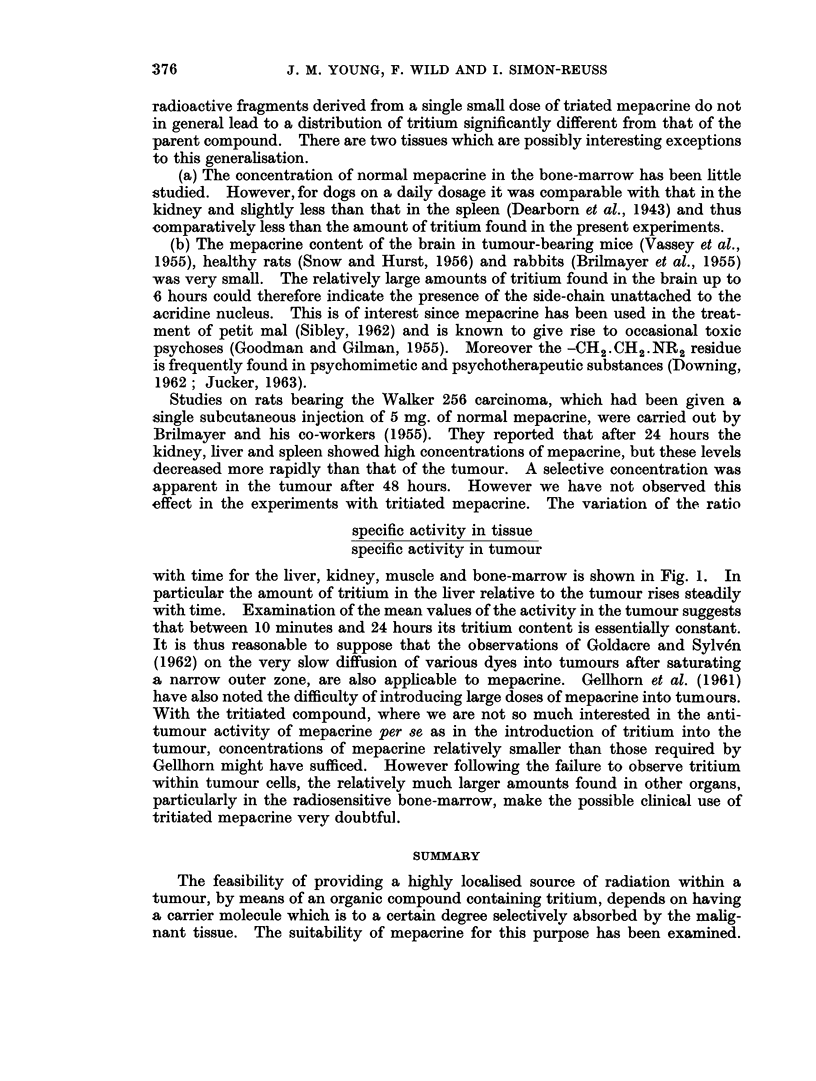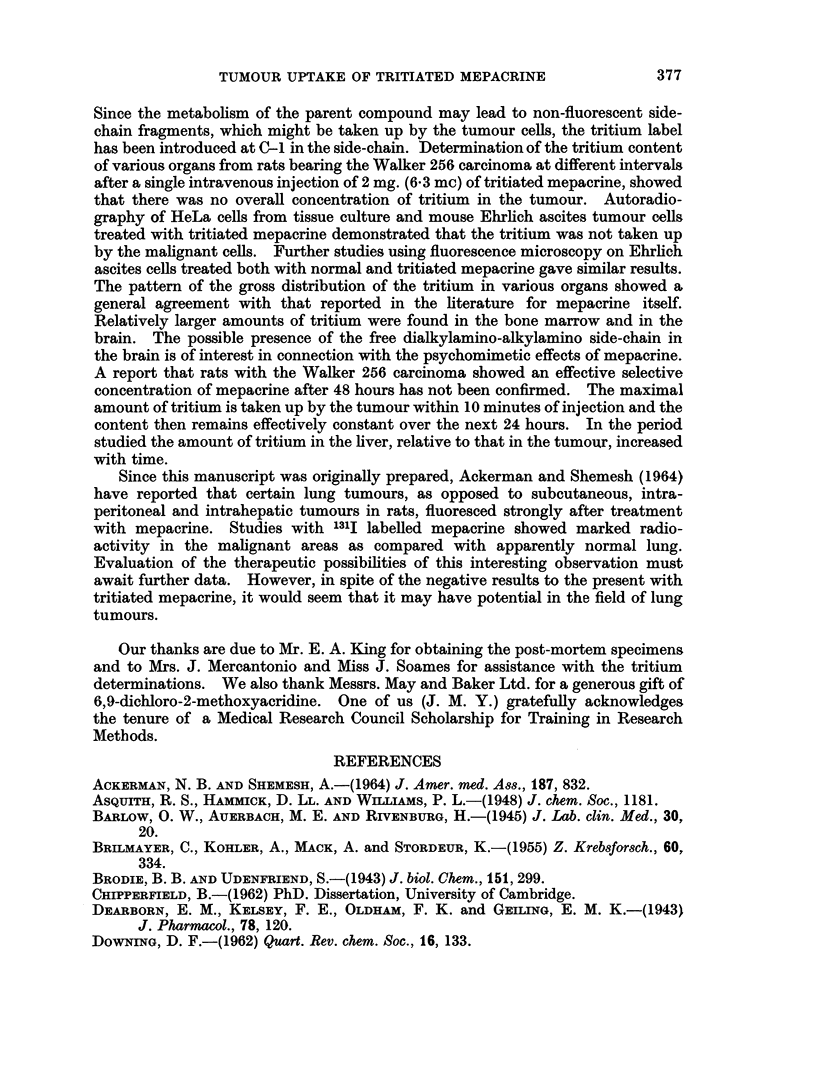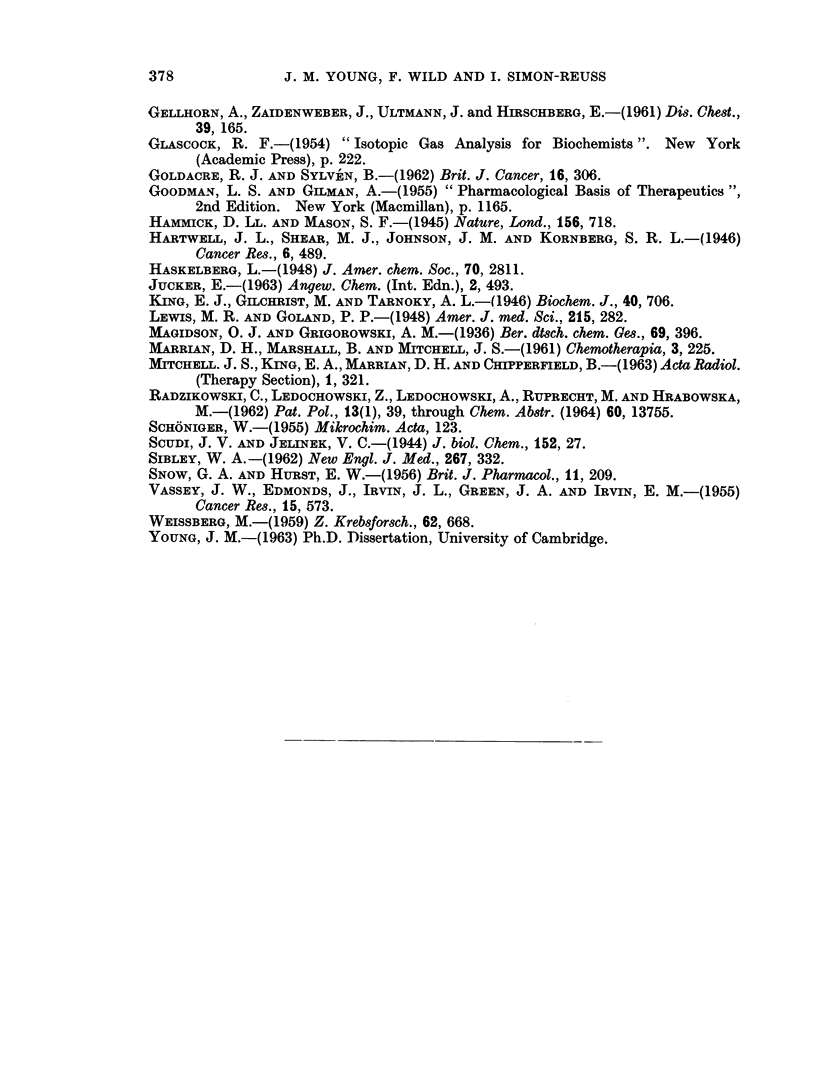# Studies of the Uptake of Tritium Labelled Mepacrine by Tumours

**DOI:** 10.1038/bjc.1965.44

**Published:** 1965-06

**Authors:** J. M. Young, F. Wild, I. Simon-Reuss


### STUDIES OF THE UPTAKE OF TRITIUM LABELLED MEPACRINE BY TUMOURS

J. M. YOUNG, F. WILD AND I. SIMON-REUSS

*From the Department of Medicine and the Department of Radiotherapeutics University of Cambridge, Tennis Court Road, Cambridge*

Received for publication December 2, 1964

THE radiotherapy of advanced and inoperable tumours by the internal administration of organic compounds labelled with radioactive isotopes, has been a special interest of Mitchell and his co-workers in this department (Marrian, Marshall and Mitchell, 1961 ; Mitchell *et al.*, 1963). Tritium has been the radioactive isotope of choice by reason of the very small mean path length, and hence the highly localised effect, of its $\beta$ particle in tissues. The feasibility of this approach depends on having an organic molecule which shows a certain degree of selective uptake into the malignant cells. During the course of these investigations we have examined the suitability of mepacrine (6-chloro-9-(4-diethylamino-1-methylbutylamino)-2-methoxyacridine) as a carrier for the tritium.

Evidence that normal mepacrine shows a limited carcinostatic activity has been obtained by several groups (Hartwell *et al.*, 1946 ; Lewis and Goland, 1948 ; Vassey *et al.*, 1955 ; Radzikowski *et al.*, 1962). Recently Gellhorn and his co-workers (1961) have shown that anti-tumour effects can be obtained provided that a sufficiently high concentration of the drug can be introduced into the tumour. On this basis they have demonstrated mepacrine to be useful clinically for the control of neoplastic effusions. The mode by which mepacrine exerts this action remains unknown and it has not been established whether the compound itself or a metabolite is the active entity. There have been suggestions however, that this effect is associated with a selective concentration of the compound in the malignant tissue. Thus mepacrine administered to mice bearing a transplanted sarcoma or a spontaneous mammary gland carcinoma was reported to lead to a selective staining of the tumour (Lewis and Goland, 1948). This effect was not confirmed by a subsequent quantitative study (Vassey *et al.*, 1955). However, in 44 out of 51 biopsies of tumours from patients treated with mepacrine, the concentration of the compound in the malignant tissue was found to be higher than in what was considered to be a normal part of the same organ (Brilmayer *et al.*, 1955). The same group also reported that tumour-bearing rats, injected with mepacrine, showed an overall selective uptake in the tumour after 48 hours. In rabbits, however, the primary tumour showed relatively little uptake and attention was called to the problem of comparing animal and human tumours, in particular where the former become necrotic very much more rapidly than the latter. In later qualitative studies on animal tumours, using fluorescence microscopy, Weissberg (1959) observed that the fluorescence, and hence the mepacrine, occurred only irregularly or not at all in the tumour. Further, there was no evidence of the fluorescence within the tumour cells.

In some circumstances, however, mepacrine may undergo considerable degradation *in vivo*. Thus after two weeks on a diet containing mepacrine, dogs showed no evidence of further storage or greater elimination, yet only 4 % of the daily intake could be detected in the urine (Dearborn *et al.*, 1943). Scudi and Jelinek (1944) also observed the urinary excretion in dogs to be complex. Although the problem of the metabolism of mepacrine has been examined by several groups (Scudi and Jelinek, 1944; Hammick and Mason, 1945; King, Gilchrist and Tarnoky, 1946), the metabolites are still largely unknown. Of those which have been identified, two have lost the side-chain (Hammick and Mason, 1945). Thus the observation, recently made by us, that mepacrine reacts under mild conditions *in vitro* with thiols, leading to the separation of the mepacrine side-chain (Young and Wild, unpublished; Young, 1963), may be relevant here. Full experimental details and a discussion of this reaction will be presented elsewhere. With simple thiols the products of the reaction are analogous to those found with hydrogen sulphide (Asquith, Hammick and Williams, 1948), but in the case of cysteine and glutathione this simple pattern is not followed, although the side-chain is still split off. Thus cysteine, incubated with mepacrine at 37° C. in phosphate buffer, pH 7·1, for 48 hours, forms the insoluble *N*, *S*-bis(6-chloro-2-methoxy-9-acridinyl)cysteine in about 50 % yield. Although this reaction with cysteine and glutathione has not been investigated *in vivo*, it is conceivable that where concentrations of mepacrine are present for a length of time it may be significant. Thus some knowledge of the fate of the side-chain is important in any consideration of the mepacrine as a carrier for the tritium. An alteration in the nature of the molecule bearing the tritium is no disadvantage, if the new carrier goes into, or remains in, the tumour. Whereas, in general, metabolites containing the acridine nucleus will probably be fluorescent, the non-fluorescent side-chain will not have been detected either by fluorescence microscopy or by the methods used to estimate unchanged mepacrine. It should be noted however, that the acridine derivatives formed by the reaction of mepacrine with cysteine and glutathione are only very weakly fluorescent. We have therefore prepared mepacrine labelled with high specific activity with tritium at C–1 in the side-chain, and have examined the distribution of the tritium in rats bearing the Walker 256 carcinoma. To provide the optimum therapeutic dose of radiation, the localisation of the tritium within the tumour cells is necessary, since the mean range of the tritium $\beta$ particle in unit density tissue (1 $\mu$) is much smaller than the diameter of most human cells. The uptake of the tritium by HeLa cells in tissue culture and mouse Ehrlich ascites cells *in vivo* has therefore been examined by autoradiography.

### MATERIALS AND METHODS

### *Preparation of tritium labelled mepacrine dihydrochloride*

This has been prepared in two stages from 1-diethylaminopentan-4-one using a small scale adaptation of the method of Haskelberg (1948) for the first stage, and that of Magidson and Grigorowski (1936) for the second. The intermediate, 4-diethylamino-1-methylbutylamine, was not isolated.

A solution of 1-diethylaminopentan-4-one (0·033 ml.) in anhydrous ammoniacal ethanol (0·3 ml., 15 % w/v), containing Raney nickel as catalyst, was stirred in an atmosphere of tritium gas (1 curie, i.e. 0·4 ml. at n.t.p.) in a microhydrogenation apparatus similar to that of Glascock (1954). After 24 hours hydrogen gas was admitted and stirring continued until no further uptake occurred. The catalyst was removed from the reaction mixture by centrifugation and the supernatant concentrated *in vacuo* to small volume. To this solution 6,9-dichloro-2-methoxy-acridine (0·035 g.) and phenol (c. 0·2 g.) were added and the stirred mixture heated at 90° for 2½ hours. The resulting gold syrup was partitioned between benzene and 20 % aqueous sodium hydroxide, and the benzene layer applied to an alumina column. Elution with benzene led to the slow separation of several fluorescent byproducts, the major of these being 6-chloro-2-methoxy-9-phenoxyacridine. The mepacrine base remaining adsorbed on the column was eluted with benzene/ether (1 : 1) and converted into the hydrochloride using dry hydrogen chloride gas. Yield 0·018 g. (20 % overall), m.p. 246–8°. A sample of normal mepacrine hydrochloride had m.p. 248–50° and an identical u.v. spectrum (0·5 N hydrochloric acid). In two preparations the tritiated mepacrine dihydrochloride had a specific activity of 1·2 curies/millimole (2·26 mc/mg.) and 1·6 c/mM (3·2 mc/mg.). Paper chromatography (see under " position of the label ") showed one spot only with which virtually all the radioactivity was associated. An 8 week old sample had the same electrophoretic mobility (see under " Stability ") as a control of normal mepacrine, and had 98·6 % of the radioactivity associated with it.

*Position of the label*

This has been confirmed by treatment of the tritiated mepacrine with hydrogen sulphide, which leads to the removal of the side-chain (Asquith, Hammick and Williams, 1948).

Through a solution of isotopically diluted (*c.* 1 : 150) tritiated mepacrine (5 mg.) in ethanol/" 880 " ammonia (4 ml., 1 : 3), hydrogen sulphide was passed for 1½ hours. The small amount of 6-chloro-2-methoxyacridan-9-thione which separated was centrifuged off. Chromatograms (silicic acid impregnated Whatman 3 MM paper, *n*-butanol/acetic acid/water, 4 : 1 : 5) of the supernatant scanned for (a) radioactivity ($R_F$ 0·23), (b) fluorescence and u.v. absorption ($R_F$ 0·94) and (c) ninhydrin positive products ($R_F$ 0·23), compared with a control of the tritiated mepacrine (fluorescence and radio-activity, $R_F$ 0·64), demonstrated that more than 99 % of the radio-activity resided with the side-chain.

*Liability to exchange*

Tritiated mepacrine, diluted with normal mepacrine (*c.* 1 : 150), was dissolved in distilled water and 6 % sulphuric acid. After 8 weeks at room temperature, the total activity of the solutions was compared with the activity which could be extracted into benzene after the addition of alkali. The tritium label was thus shown to be at least 94 % stable to proton exchange with the solvent water. This figure may be low since (a) mepacrine base is strongly adsorbed on to glass surfaces (Brodie and Udenfriend, 1943) and (b) slight hydrolysis of the mepacrine during the 8 weeks may have produced water-soluble tritiated products.

*Stability.*—Electrophoresis (10 % acetic acid, Whatman 3 MM paper) and chromatography of an 8 week old sample of the tritiated mepacrine dihydrochloride, which had been stored as the solid in the dark at − 25° C., showed the presence of very small amounts of 6-chloro-2-methoxyacridone. This compound was not present on chromatograms of the freshly prepared material, and further chromatograms showed that the amount present increased with time. As normal mepacrine dihydrochloride is stable under these conditions, this decomposition is considered to be due to self-irradiation.

*Distribution of the tritium in tumour-bearing rats*

Five rats bearing an 8 day old Walker 256 carcinoma were injected in the tail vein with 0·5 ml. of a normal saline solution containing 2 mg. tritiated mepacrine dihydrochloride (6·3 mc tritium). The animals were killed at intervals of 10 minutes, 1, 6, 24, and 48 hours, and the specimens stored at − 25° C. until required for counting. The tritium content of various tissues was determined using a modification of the Schöniger (1955) oxygen flask combustion technique (Chipperfield, 1962). Tissue samples of about 20 mg. wet weight are combusted in an oxygen enriched atmosphere in the presence of normal non-active water. The tritiated water vapour formed is absorbed in the large excess of normal water, and can then be determined by liquid scintillation counting. The accuracy of this method is about ± 5 %.

*Uptake of the tritium into tumour cells*

HeLa cells in tissue culture were treated with a $2 \times 10^{-5}$M solution of tritiated mepacrine dihydrochloride for 24 and 48 hours. Smears were then examined by autoradiography.

Mice with the Ehrlich ascites tumour were injected intraperitoneally with 1 mg. tritiated mepacrine dihydrochloride (2·26 mc/mg.) and killed after 3, 7, 24 and 30 hours. A second group were treated with 2 mg. of the tritiated material and killed at 0, 5, 24, 30 and 48 hours. Smears from the tumour were examined by fluorescence microscopy and autoradiography. Two further groups were treated as above, but with normal commercial mepacrine, and the post-mortem specimens examined for visible staining and fluorescence.

### RESULTS

*Distribution of tritium in rats bearing the Walker carcinoma*

The specific activity of various tissues from the rats killed at intervals is set out in Table I. Each figure is the average of the values obtained from 2–4 different samples of the same tissue. The agreement between these samples varied markedly from tissue to tissue. Thus while the values for the liver were within 7·5 % of the mean, those from the tumour consistently showed considerable

TABLE I.—*Activities in Tissues of Rats Following a Single I.V. Injection of Tritiated Mepacrine*

All values in mμc/mg.

Each figure is the mean of 2–4 determinations on the same animal

| | 10 min. | 1 hr. | 6 hr. | 24 hr. | 48 hr. |
|---|---|---|---|---|---|
| Tumour | 17 | 15 | 18 | 13 | 5·8 |
| Muscle | 13 | 34 | 19 | 10 | 4·4 |
| Kidney | 150 | 138 | 117 | 50 | 34 |
| Blood | 5·0 | 1·9 | 1·4 | 0·7† | 0·8 |
| Bone marrow | 34 | 87 | 145* | 81* | 108* |
| Brain | 29 | 31 | 17 | 5·4 | 1·8 |
| Testis | 3·5 | 5·0 | 4·8 | 5·4 | 3·9 |
| Liver | 36 | 64 | 115 | 105 | 60 |
| Spleen | 42 | 68 | 64 | 82 | 39 |
| Gut | 43 | 61 | 28 | 31 | 10 |

* One determination only.

† Unreliable value.

variation—individual values differing from each other by as much as a factor of 3·5. Of the other tissues only the determinations on the brain showed a consistently large variability, up to a factor of 2. This variability must be considered to represent an uneven distribution of tritium in the organ. In the case of the tumour this is not unduly surprising since the difficulty of the penetration of dyes into the tumour mass, with a consequently dye-poor area in the necrotic centre, has been demonstrated by Goldacre and Sylvén (1962).

The amount of bone marrow extracted from three of the rats was sufficient for only one determination in each case. The values obtained show a reasonable consistency from rat to rat, and the large factor between the activity of the 48 hour bone-marrow specimen and the mean value for the tumour is far beyond experimental error.

A comparison of the rate of uptake and initial loss of tritium from the tumour and certain other tissues is shown in Fig. 1. The ratio

$$\frac{\text{specific activity of the tissue}}{\text{specific activity of the tumour}}$$

has been used to meet the problem of the variability of individual animals in such features as weight and possibly slightly different doses.

*Uptake of the tritium into tumour cells*

Autoradiographs of monolayer HeLa cells, treated with a $2 \times 10^{-5}$ M solution of tritiated mepacrine dihydrochloride for 24 and 48 hours, showed no evidence of any uptake of tritium within the cells. A more concentrated solution of normal mepacrine ($4 \times 10^{-4}$ M) produced a general toxic effect on the culture.

Autoradiographs of air-dried smears of Ehrlich ascites tumour cells, taken from mice treated (IP) with 1 mg. and 2 mg. tritiated mepacrine and killed at various intervals, showed no uptake of the tritium into the tumour cells at any time. With the same doses of normal mepacrine no staining effects were observed. Mice killed immediately after injection (IP) with 2 mg. tritiated or normal mepacrine showed fluorescence only in the blood. In mice killed after 5 hours, about 2% of the tumour cells appeared to fluoresce but no fluorescence was observed after longer periods.

FIG. 1.

### DISCUSSION

It is clear from the activities of various tissues, set out in Table I, that at no time is there an overall selective concentration of tritium in the tumour. After both 24 and 48 hours, 5 out of the 9 tissues examined showed a higher tritium concentration than that of the tumour. Most important from one hour onwards the radiosensitive bone-marrow was much more active than the tumour. Notwithstanding the reservations as to the accuracy of the measurements on the bone-marrow, the factor of 20 after 48 hours is far beyond experimental error. Further, no evidence was found for any uptake of the tritium label into HeLa cells in tissue culture or mouse Ehrlich ascites tumour cells *in vivo*. Hence the prospects for tritiated mepacrine as a drug which is to provide selective irradiation of the tumour, appear slight. The failure to observe any fluorescence within the ascites cells after treatment with normal mepacrine, apart from a few cells after 5 hours, confirms the observations of Weissberg (1959). Thus non-fluorescent radioactive fragments, formed by metabolic degradation, are either produced in very small amounts or, like the parent compound, are not taken up by the cells.

Quantitative values for the distribution of normal mepacrine in tumour-bearing mice have been reported by Vassey and his co-workers (1955). The mepacrine was administered over a period of time in the food, but nevertheless there is general agreement with the results from the tritiated compound, the relative concentrations of mepacrine and tritium being liver > spleen > kidney > tumour > testis > blood. The same pattern, excluding the tumour, has been observed in healthy animals (Barlow, Auerbach and Rivenburg, 1945; Snow and Hurst, 1956). Thus although under certain circumstances considerable degradation of mepacrine may occur *in vivo* (Dearborn *et al.*, 1943; Scudi and Jelinek, 1944), any radioactive fragments derived from a single small dose of triated mepacrine do not in general lead to a distribution of tritium significantly different from that of the parent compound. There are two tissues which are possibly interesting exceptions to this generalisation.

(a) The concentration of normal mepacrine in the bone-marrow has been little studied. However, for dogs on a daily dosage it was comparable with that in the kidney and slightly less than that in the spleen (Dearborn *et al.*, 1943) and thus comparatively less than the amount of tritium found in the present experiments.

(b) The mepacrine content of the brain in tumour-bearing mice (Vassey *et al.*, 1955), healthy rats (Snow and Hurst, 1956) and rabbits (Brilmayer *et al.*, 1955) was very small. The relatively large amounts of tritium found in the brain up to 6 hours could therefore indicate the presence of the side-chain unattached to the acridine nucleus. This is of interest since mepacrine has been used in the treatment of petit mal (Sibley, 1962) and is known to give rise to occasional toxic psychoses (Goodman and Gilman, 1955). Moreover the $-CH_2.CH_2.NR_2$ residue is frequently found in psychomimetic and psychotherapeutic substances (Downing, 1962 ; Jucker, 1963).

Studies on rats bearing the Walker 256 carcinoma, which had been given a single subcutaneous injection of 5 mg. of normal mepacrine, were carried out by Brilmayer and his co-workers (1955). They reported that after 24 hours the kidney, liver and spleen showed high concentrations of mepacrine, but these levels decreased more rapidly than that of the tumour. A selective concentration was apparent in the tumour after 48 hours. However we have not observed this effect in the experiments with tritiated mepacrine. The variation of the ratio

$$\frac{\text{specific activity in tissue}}{\text{specific activity in tumour}}$$

with time for the liver, kidney, muscle and bone-marrow is shown in Fig. 1. In particular the amount of tritium in the liver relative to the tumour rises steadily with time. Examination of the mean values of the activity in the tumour suggests that between 10 minutes and 24 hours its tritium content is essentially constant. It is thus reasonable to suppose that the observations of Goldacre and Sylvén (1962) on the very slow diffusion of various dyes into tumours after saturating a narrow outer zone, are also applicable to mepacrine. Gellhorn *et al.* (1961) have also noted the difficulty of introducing large doses of mepacrine into tumours. With the tritiated compound, where we are not so much interested in the anti-tumour activity of mepacrine *per se* as in the introduction of tritium into the tumour, concentrations of mepacrine relatively smaller than those required by Gellhorn might have sufficed. However following the failure to observe tritium within tumour cells, the relatively much larger amounts found in other organs, particularly in the radiosensitive bone-marrow, make the possible clinical use of tritiated mepacrine very doubtful.

### SUMMARY

The feasibility of providing a highly localised source of radiation within a tumour, by means of an organic compound containing tritium, depends on having a carrier molecule which is to a certain degree selectively absorbed by the malignant tissue. The suitability of mepacrine for this purpose has been examined.

Since the metabolism of the parent compound may lead to non-fluorescent side-chain fragments, which might be taken up by the tumour cells, the tritium label has been introduced at C–1 in the side-chain. Determination of the tritium content of various organs from rats bearing the Walker 256 carcinoma at different intervals after a single intravenous injection of 2 mg. (6·3 mc) of tritiated mepacrine, showed that there was no overall concentration of tritium in the tumour. Autoradiography of HeLa cells from tissue culture and mouse Ehrlich ascites tumour cells treated with tritiated mepacrine demonstrated that the tritium was not taken up by the malignant cells. Further studies using fluorescence microscopy on Ehrlich ascites cells treated both with normal and tritiated mepacrine gave similar results. The pattern of the gross distribution of the tritium in various organs showed a general agreement with that reported in the literature for mepacrine itself. Relatively larger amounts of tritium were found in the bone marrow and in the brain. The possible presence of the free dialkylamino-alkylamino side-chain in the brain is of interest in connection with the psychomimetic effects of mepacrine. A report that rats with the Walker 256 carcinoma showed an effective selective concentration of mepacrine after 48 hours has not been confirmed. The maximal amount of tritium is taken up by the tumour within 10 minutes of injection and the content then remains effectively constant over the next 24 hours. In the period studied the amount of tritium in the liver, relative to that in the tumour, increased with time.

Since this manuscript was originally prepared, Ackerman and Shemesh (1964) have reported that certain lung tumours, as opposed to subcutaneous, intraperitoneal and intrahepatic tumours in rats, fluoresced strongly after treatment with mepacrine. Studies with $^{131}I$ labelled mepacrine showed marked radioactivity in the malignant areas as compared with apparently normal lung. Evaluation of the therapeutic possibilities of this interesting observation must await further data. However, in spite of the negative results to the present with tritiated mepacrine, it would seem that it may have potential in the field of lung tumours.

Our thanks are due to Mr. E. A. King for obtaining the post-mortem specimens and to Mrs. J. Mercantonio and Miss J. Soames for assistance with the tritium determinations. We also thank Messrs. May and Baker Ltd. for a generous gift of 6,9-dichloro-2-methoxyacridine. One of us (J. M. Y.) gratefully acknowledges the tenure of a Medical Research Council Scholarship for Training in Research Methods.

### REFERENCES

ACKERMAN, N. B. AND SHEMESH, A.—(1964) *J. Amer. med. Ass.*, **187**, 832.
ASQUITH, R. S., HAMMICK, D. LL. AND WILLIAMS, P. L.—(1948) *J. chem. Soc.*, 1181.
BARLOW, O. W., AUERBACH, M. E. AND RIVENBURG, H.—(1945) *J. Lab. clin. Med.*, **30**, 20.
BRILMAYER, C., KOHLER, A., MACK, A. and STORDEUR, K.—(1955) *Z. Krebsforsch.*, **60**, 334.
BRODIE, B. B. AND UDENFRIEND, S.—(1943) *J. biol. Chem.*, **151**, 299.
CHIPPERFIELD, B.—(1962) PhD. Dissertation, University of Cambridge.
DEARBORN, E. M., KELSEY, F. E., OLDHAM, F. K. and GEILING, E. M. K.—(1943) *J. Pharmacol.*, **78**, 120.
DOWNING, D. F.—(1962) *Quart. Rev. chem. Soc.*, **16**, 133.

GELLHORN, A., ZAIDENWEBER, J., ULTMANN, J. and HIRSCHBERG, E.—(1961) *Dis. Chest.*, **39**, 165.
GLASCOCK, R. F.—(1954) "Isotopic Gas Analysis for Biochemists". New York (Academic Press), p. 222.
GOLDACRE, R. J. AND SYLVÉN, B.—(1962) *Brit. J. Cancer*, **16**, 306.
GOODMAN, L. S. AND GILMAN, A.—(1955) "Pharmacological Basis of Therapeutics", 2nd Edition. New York (Macmillan), p. 1165.
HAMMICK, D. LL. AND MASON, S. F.—(1945) *Nature, Lond.*, **156**, 718.
HARTWELL, J. L., SHEAR, M. J., JOHNSON, J. M. AND KORNBERG, S. R. L.—(1946) *Cancer Res.*, **6**, 489.
HASKELBERG, L.—(1948) *J. Amer. chem. Soc.*, **70**, 2811.
JUCKER, E.—(1963) *Angew. Chem.* (Int. Edn.), **2**, 493.
KING, E. J., GILCHRIST, M. AND TARNOKY, A. L.—(1946) *Biochem. J.*, **40**, 706.
LEWIS, M. R. AND GOLAND, P. P.—(1948) *Amer. J. med. Sci.*, **215**, 282.
MAGIDSON, O. J. AND GRIGOROWSKI, A. M.—(1936) *Ber. dtsch. chem. Ges.*, **69**, 396.
MARRIAN, D. H., MARSHALL, B. AND MITCHELL, J. S.—(1961) *Chemotherapia*, **3**, 225.
MITCHELL. J. S., KING, E. A., MARRIAN, D. H. AND CHIPPERFIELD, B.—(1963) *Acta Radiol.* (Therapy Section), **1**, 321.
RADZIKOWSKI, C., LEDOCHOWSKI, Z., LEDOCHOWSKI, A., RUPRECHT, M. AND HRABOWSKA, M.—(1962) *Pat. Pol.*, **13**(1), 39, through *Chem. Abstr.* (1964) **60**, 13755.
SCHÖNIGER, W.—(1955) *Mikrochim. Acta*, 123.
SCUDI, J. V. AND JELINEK, V. C.—(1944) *J. biol. Chem.*, **152**, 27.
SIBLEY, W. A.—(1962) *New Engl. J. Med.*, **267**, 332.
SNOW, G. A. AND HURST, E. W.—(1956) *Brit. J. Pharmacol.*, **11**, 209.
VASSEY, J. W., EDMONDS, J., IRVIN, J. L., GREEN, J. A. AND IRVIN, E. M.—(1955) *Cancer Res.*, **15**, 573.
WEISSBERG, M.—(1959) *Z. Krebsforsch.*, **62**, 668.
YOUNG, J. M.—(1963) Ph.D. Dissertation, University of Cambridge.